# Unrecorded alcohol consumption in Lithuania: a modelling study for 2000–2021

**DOI:** 10.1093/alcalc/agad063

**Published:** 2023-10-07

**Authors:** Mindaugas Štelemėkas, Nijolė Goštautaitė Midttun, Shannon Lange, Vaida Liutkutė-Gumarov, Jakob Manthey, Laura Miščikienė, Janina Petkevičienė, Ričardas Radišauskas, Jürgen Rehm, Justina Trišauskė, Tadas Telksnys, Mark James Thompson

**Affiliations:** Health Research Institute, Faculty of Public Health, Lithuanian University of Health Sciences, Tilžės str.18, Kaunas 47181, Lithuania; Department of Preventive Medicine, Faculty of Public Health, Lithuanian University of Health Sciences, Tilžės str.18, Kaunas 47181, Lithuania; Lithuanian Tobacco and Alcohol Control Coalition, Stiklių st. 8, Vilnius 01131, Lithuania; Lithuanian Tobacco and Alcohol Control Coalition, Stiklių st. 8, Vilnius 01131, Lithuania; Mental Health Initiative, Teatro st. 3-10, Vilnius 03107, Lithuania; Institute for Mental Health Policy Research, Centre for Addiction and Mental Health, 33 Ursula Franklin Street, Toronto, ON M5S2S1, Canada; Campbell Family Mental Health Research Institute, Centre for Addiction and Mental Health, 250 College Street, Toronto, ON M5T 1R8, Canada; Department of Psychiatry, University of Toronto, 250 College Street, 8thfloor, Toronto, ON M5T1R8, Canada; Health Research Institute, Faculty of Public Health, Lithuanian University of Health Sciences, Tilžės str.18, Kaunas 47181, Lithuania; Lithuanian Tobacco and Alcohol Control Coalition, Stiklių st. 8, Vilnius 01131, Lithuania; Department of Psychiatry and Psychotherapy, Center for Interdisciplinary Addiction Research, University Medical Center Hamburg-Eppendorf, Hamburg 20246, Germany; Department of Psychiatry, Medical Faculty, University of Leipzig, Semmelweisstraße 10, Leipzig 04103,Germany; Health Research Institute, Faculty of Public Health, Lithuanian University of Health Sciences, Tilžės str.18, Kaunas 47181, Lithuania; Health Research Institute, Faculty of Public Health, Lithuanian University of Health Sciences, Tilžės str.18, Kaunas 47181, Lithuania; Department of Preventive Medicine, Faculty of Public Health, Lithuanian University of Health Sciences, Tilžės str.18, Kaunas 47181, Lithuania; Institute of Cardiology, Lithuanian University of Health Sciences, Sukilėlių pr. 15, Kaunas 50103, Lithuania; Department of Environmental and Occupational Medicine, Faculty of Public Health, Lithuanian University of Health Sciences, Tilžės str.18, Kaunas 47181, Lithuania; Institute for Mental Health Policy Research, Centre for Addiction and Mental Health, 33 Ursula Franklin Street, Toronto, ON M5S2S1, Canada; Campbell Family Mental Health Research Institute, Centre for Addiction and Mental Health, 250 College Street, Toronto, ON M5T 1R8, Canada; Department of Psychiatry, University of Toronto, 250 College Street, 8thfloor, Toronto, ON M5T1R8, Canada; Department of Psychiatry and Psychotherapy, Center for Interdisciplinary Addiction Research, University Medical Center Hamburg-Eppendorf, Hamburg 20246, Germany; Dalla Lana School of Public Health, Institute of Health Policy, Management and Evaluation, University of Toronto, 155 College Street, Toronto, ON M5T1P8, Canada; Program on Substance Abuse, Public Health Agency of Catalonia, 81-95 Roc Boronat St., Barcelona 08005, Spain; Institute of Medical Science, Faculty of Medicine, University of Toronto, Medical Sciences Building, 1 King’s College Circle, Room 2374, Toronto, ON M5S1A8, Canada; Health Research Institute, Faculty of Public Health, Lithuanian University of Health Sciences, Tilžės str.18, Kaunas 47181, Lithuania; Health Research Institute, Faculty of Public Health, Lithuanian University of Health Sciences, Tilžės str.18, Kaunas 47181, Lithuania; Health Research Institute, Faculty of Public Health, Lithuanian University of Health Sciences, Tilžės str.18, Kaunas 47181, Lithuania; Institute for Wealth and Asset Management, Zurich University of Applied Sciences, Winterthur, Switzerland

**Keywords:** alcohol, unrecorded alcohol, alcohol consumption, Lithuania

## Abstract

The aim of the study was to estimate unrecorded alcohol consumption in Lithuania for the period 2000–2021 using an indirect method for modelling consumption based on official consumption data and indicators of alcohol-related harm. Methodology employed for estimating the unrecorded alcohol consumption was proposed by Norström and was based on the country’s 2019 European Health Interview Survey and indicators of fully alcohol-attributable mortality. The proportion of unrecorded alcohol consumption was estimated as 8.30% (95% CI 7.7–8.9%) for 2019 in Lithuania. The estimated total (recorded and unrecorded) alcohol per capita consumption among individuals 15 years of age and older in 2019 was 12.2 L of pure alcohol, 1.01 (95% CI 0.94–1.09%) L of which is likely unrecorded. The lowest unrecorded alcohol level was estimated for 2009 and 2014, while 2018 had the highest level (i.e. 9.33% of total alcohol per capita consumption). Unrecorded alcohol consumption in Lithuania is likely to be modest when compared to recorded alcohol consumption, the latter of which still remains a major challenge to public health.

## Teaser

**Short Summary:** This paper presents estimates of unrecorded alcohol consumption in Lithuania, based on historical series and on data from the 2019 European Health Interview Survey. Unrecorded alcohol consumption in Lithuania is likely to be relatively modest, suggesting that recorded alcohol consumption remains by far the major contributor to alcohol-attributable harm in Lithuania.

## Background

The highest levels of alcohol per capita (APC) consumption are observed in countries of the World Health Organization (WHO) European Region, especially in countries in Eastern Europe. In 2016, Lithuania, member of the European Union (EU) with a population of 2.8 million, had one of the highest total APC consumption in litres of pure alcohol among individuals 15 years of age and older (WHO 2018). The publication of the WHO data has led to a lot of public debate in all circles of society and structures at the time. It has also created public controversy over the accuracy of the consumption estimates (LRT 2017), largely due to a discrepancy between recorded APC consumption provided at the time by Statistics Lithuania (which was 13.2 L of APC in 2016) and total APC consumption provided by the WHO, which included both recorded and unrecorded (16.3 L of APC in 2016). Therefore, the analysis of unrecorded alcohol consumption is important to Lithuania as to any country undergoing the alcohol policy debate as alcohol industry may use any level of uncertainty on the matter as an argument against alcohol control policy changes, especially when discussing the taxation. (Zatonski *et al*. 2018, Sama *et al*. 2021).

Rehm and colleagues (Rehm *et al.* 2022a) state that recorded alcohol consumption includes taxed and otherwise registered alcoholic beverages supplied to the market/consumed, which are accounted for in national statistics. In contrast, unrecorded alcohol consumption is a much broader category that may include legally obtained products (homemade, medical products for human consumption, alcohol from cross-border shopping), surrogate alcohol, which is not officially intended for human consumption (non-beverage medicinal or industrial alcohol, such like cosmetics, mouthwash, denatured alcohol, etc.), but also (illegally) homemade or illegally produced alcohol on an industrial scale—so called ‘third shift’ alcohol (alcohol beverages produced, but not registered in a production facility).

In the early 2000s, Lithuanian legislature failed to introduce a timely policy response to the increasing alcohol-related harms (Grabauskas *et al.* 2009). Increasing levels of APC consumption and corresponding alcohol-related harm ultimately led to a policy response in the Year 2007. Since then, a number of alcohol control measures were adopted (Miščikienė *et al.* 2020, Stumbrys *et al.* 2020, Štelemėkas *et al.* 2021). These measures seem to have had an effect: the official recorded pure APC consumption declined to 11.1 L in 2019; alcohol-related disease has declined slightly, but confounded with potential comorbidity interactions with COVID-19 (Institute of Hygiene 2022). Consumption volume is up from 8.0 L per capita in 1998, but less than peak consumption in 2011 of 14.7 L per capita. Collection methodologies and the composition of recorded and unrecorded consumption could draw a more clear conclusion about the trend, as well as support the analysis of the impact of control measures on alcohol-attributable harm, with the Lithuanian natural experiment in alcohol control policies as a case study (Babor *et al.* 2023).

Lithuania became a member of the EU in 2004 and joined the Schengen Area in 2007, which has led to economic benefits as well as unrestrictive travel throughout the EU for its citizens. Although positive in terms of economic development, access to the European common market also created a challenge of tax evasion from cross-border shopping when alcohol is legally brought across the border but registered in another jurisdiction. The studies of unrecorded alcohol in Lithuania for many years remained fragmented (Health Research Institute 2018), and there was a lack of any systematic data collection aimed at capturing the volume of unrecorded alcohol consumption. However, a survey question on unrecorded alcohol consumption was included in the Lithuanian version of the European Health Interview Survey (EHIS) in 2019 (Eurostat 2020, Statistics Lithuania 2020) in addition to the standard questions of alcohol consumption. The EHIS study is the only routine survey in Lithuania across a wide range of the population to monitor health risk behaviours such as drinking habits and unrecorded consumption. The survey data on alcohol consumption are a standard approach used to estimate the level of unrecorded alcohol consumption for a given year, which can be extended by the indirect methods to model the level of unrecorded consumption in a multiple year timeline (Norström 1998).

According to Manthey and colleagues (Manthey *et al.* 2020), high-income countries, with the exception of Estonia, Finland, and Sweden, often are missing questions related to unrecorded alcohol consumption in their national surveys. However, some international surveys like the Standardised European Alcohol Survey developed by the Joint Action on Reducing Alcohol Related Harm (RARHA SEAS) (Moskalewicz *et al.* 2016) may contribute to improved monitoring of the levels of unrecorded alcohol consumption. Data from RARHA SEAS also highlight the fact that unrecorded alcohol consumption is prevalent in high-income countries and should be monitored. It may be particularly important to track unrecorded alcohol consumption over time in countries that have implemented some evidence-based alcohol policies, bearing in mind that some object to policies, such as increasing taxation, on the grounds that they give rise to increases in unrecorded APC consumption. Given the lack of estimates on unrecorded alcohol consumption and Lithuania’s progress in implementing strong alcohol policies, we aimed to estimate unrecorded alcohol consumption in Lithuania for 2000–2021. To achieve this, we modelled the trend of unrecorded alcohol consumption based on the 2019 EHIS survey data and alcohol-attributable mortality trends. To our knowledge, this is the first national attempt to define long-term unrecorded alcohol consumption data.

## Methods

### Lithuanian European Health Interview Survey data and proportion of unrecorded alcohol per capita in 2019

The national statistics office, Statistics Lithuania, performed the study in the fall of 2019 in the framework of the EHIS (EHIS 2018, Statistics Lithuania 2020). A random sample was drawn from the Population Register. In total, 4923 Lithuanian residents responded to survey questions (response rate 70.3%). The survey included seven questions on respondent’s habits (quantity and frequency) of alcohol consumption. The translated survey questions on alcohol are provided in the Supplement.

The survey data received from the Statistics Lithuania were provided in standard alcohol units (SAUs), where 1 SAU represents 10 g of pure alcohol. Statistics Lithuania assumes a nominal alcohol content for each beverage type, i.e. wine 12%, spirits 40%, alcohol cocktails 13%, beer and fermented beverages 5%.

For each respondent, who indicated alcohol consumption at least once a week, the average amount of alcohol (defined in SAU) consumed was determined for five beverage types (questions V1–V6 in the questionnaire, see Supplement): beer, wine, vodka and other strong drinks, alcohol cocktails, and fermented drinks (cider, mead, etc.). Due to differences in drinking profile between Monday–Friday and Saturday–Sunday, the questions were split into two distinct sections, which allowed the estimation of the following variables in each of the previously mentioned alcohol categories: ${n}_{wd}$: the number of days that a respondent consumed alcohol on average during Mondays–Fridays; ${n}_{we}$: the number of days that a respondent consumed alcohol on average during Weekends; $SA{U}_{wd}, SA{U}_{we}$: the number of SAUs that a respondent consumed on drinking occasions during Monday–Friday and Weekends, respectively. The average daily alcohol consumption is then estimated as:


(1) \begin{equation*} SAU\mathrm{average}=\frac{1}{7}\left( SA{U}_{wd}{n}_{wd}+ SA{U}_{we}{n}_{we}\right) \end{equation*}

Note that by survey design, the above assumes total (recorded and unrecorded) alcohol consumption. In an additional question, unrecorded alcohol proportion was assessed (see the Supplement, question V7): ‘*What proportion from all of your alcohol consumption over the last 12 months was made up of alcohol bought in other countries (Latvia, Poland, etc.) and brought back to Lithuania; homemade alcohol (homemade wine, moonshine) and surrogate alcohol?*’. As a reply to this question, the respondents were asked to provide a proportion (%) of every unrecorded alcohol acquired by every alcohol beverage category (beer, wine, strong beverages, cocktails, and fermented beverages) as listed in previous questions on alcohol consumption frequency and quantity.

After that, we used the measured proportions of unrecorded alcohol acquired to approximate the amount of unrecorded alcohol consumed per each respondent. From this, using Equation (1) again, but this time with total consumption values, we obtained the amount of unrecorded alcohol consumed for each respondent. Thus, the proportion of unrecorded consumption is given by $\frac{C_{ur}}{C_{ur}+{C}_r}$, where ${C}_{ur},{C}_r$ stand for total consumption of unrecorded and recorded alcohol, respectively.

### Recorded alcohol consumption data

We obtained data on the recorded APC (in litres of pure alcohol among 15 years old and older population) from the Statistics Lithuania (Statistics Lithuania 2022) which is based on the annual study implemented by Statistics Lithuania analysing the retail recorded alcohol sale data (Statistics Lithuania 2016). However, in 2016, Statistics Lithuania attempted to improve methodology (Statistics Lithuania 2016) by reflecting the tourist effect and border duty-free sales on APC. As a result, it started to subtract border duty-free sales and incoming tourist consumption from the total APC and add the estimates of outgoing tourist APC. The effect of border duty-free and overall tourist effect produced a net negative value (between −0.04 and −0.66 L per year), indicating that inbound tourists together with the border duty-free sales result in higher sales than Lithuanians consume when traveling abroad. Thus, the tourist and duty-free data were added to the officially available recorded APC indicator from Year 2010 onwards (as per Statistics Lithuania methodology). As this methodological change of subtracting tourist effect and duty-free sales affected only the estimates from 2010 onwards, by adding it back from a Year 2010, we reinstated the consistent recorded APC dataset for the entire timeline (Years 2000–2021). The data are summarized in Table 1.

**Table 1:** Recorded APC in Lithuania, 2000–2021.

Year	Consistent recorded APC used in this analysis, 15+ population, litres of pure alcohol	Border and tourist effect on APC from 2010, 15+ population, litres of pure alcohol^a^	Statistics Lithuania, recorded APC, 15+ population, litres of pure alcohol^b^
1998	8.0	N/A	8.0
1999	8.6	N/A	8.6
2000	9.7	N/A	9.7
2001	10.5	N/A	10.5
2002	11.1	N/A	11.1
2003	11.3	N/A	11.3
2004	12.2	N/A	12.2
2005	12.5	N/A	12.5
2006	13.2	N/A	13.2
2007	13.9	N/A	13.9
2008	13.9	N/A	13.9
2009	13.1	N/A	13.1
2010	13.6	−0.105	13.5
2011	14.9	−0.204	14.7
2012	15.2	−0.471	14.7
2013	15.2	−0.661	14.5
2014	14.9	−0.655	14.2
2015	14.5	−0.464	14.0
2016	13.6	−0.441	13.2
2017	12.8	−0.077	12.3
2018	11.3	−0.089	11.2
2019	11.2	−0.093	11.1
2020	11.4	−0.033	11.4
2021	12.1	−0.040	12.1

N/A—not applicable, as the methodological change by Statistics Lithuania was introduced from 2010 onwards; ^a^The effect of a change in methodology, data provided by Statistics Lithuania (Statistics Lithuania 2016); ^b^Publicly available official data as recorded APC in Lithuania (Statistics Lithuania 2022).

## Mortality data

The fully alcohol-attributable mortality indicators were used as one of the components for the indirect estimation of unrecorded alcohol consumption. Indicators may be accessed using the publicly available health database or may be requested from the Lithuanian Institute of Hygiene (Institute of Hygiene 2022).

The main analysis was based on two merged fully alcohol-attributable mortality indicators: alcoholic liver disease (ICD-10 – K70) and accidental poisoning by and exposure to alcohol (X45). Those two mortality diagnoses form the majority of all of the fully alcohol attributable mortality in Lithuania (exceeding 80% from all fully alcohol attributable cause), and are known mortality indicators to react quickly into the changes in a population due to the level of alcohol consumption or public health policies as well as remained stable across the years in ICD coding. The alternative analysis was based on adding seven additional fully alcohol-attributable mortality causes to the ones used in the main analysis: combined diagnostic categories for the fully alcohol-attributable mortality, which included: mental and behavioural disorders due to use of alcohol (F10); degeneration of nervous system due to alcohol (G31.2); alcoholic polyneuropathy (G62.1); alcoholic cardiomyopathy (I42.6); alcohol-induced chronic pancreatitis (K86.0); intentional self-poisoning by and exposure to alcohol (X65); poisoning by and exposure to alcohol, undetermined intent (Y15). The mortality data used in the analysis are summarized in Table 2.

**Table 2:** Alcohol attributable mortality used in estimating unrecorded alcohol consumption.

Year	Mortality from Alcoholic liver disease (K70) and Accidental poisoning by and exposure to alcohol (X45), cases per 100 000 population	Mortality from majority of fully alcohol attributable causes (K70, X45, F10, G31.2, G62.1, I42.6, K86.0, X65, Y15), cases per 100 000 population
1998	20.21	
1999	16.91	
2000	18.65	
2001	23.20	29.73
2002	23.42	29.25
2003	26.08	32.56
2004	25.27	32.51
2005	29.83	37.38
2006	35.84	45.38
2007	41.38	53.88
2008	35.98	46.12
2009	25.72	32.22
2010	24.03	31.09
2011	24.96	31.14
2012	23.74	30.32
2013	24.63	29.79
2014	19.74	25.13
2015	19.58	24.27
2016	17.38	22.70
2017	15.38	19.98
2018	13.60	17.69
2019	13.78	19.21
2020	18.43	24.08
2021	20.44	27.09

Note that both recorded alcohol consumption and mortality rates need to be considered not only for the period over which the unrecorded alcohol is to be estimated. Since the Norström approach (Norström 1998) uses lagged time series models, it needs to establish a baseline model which includes data points prior to the investigation period. In this case, it was Years 1998–1999, as the models had lags no higher than 2 years. For more information on the specifics of this, see section Statistical Analyses.

## Statistical analyses

The estimation of unrecorded alcohol used in this paper is based on the method proposed by Norström (1998). This methodology isolates a cause of death that can be entirely attributed to alcohol and constructs a time series model using recorded alcohol consumption as an exogenous variable. Since the death causes can only be caused by alcohol consumption, the error of this model provides insight into unrecorded alcohol consumption. Furthermore, if the unrecorded consumption is known or can be estimated for one specific year (e.g. estimated using a survey) of the analysed period, the errors can be used to estimate unrecorded consumption for the entire period (exact method described below). Thus, the method does not estimate the level of unrecorded consumption, but allows to estimate time variations in unrecorded alcohol consumption under certain assumptions. The analysis was performed using MATLAB R2014b, IBM SPSS Statistics 27, and R version 4.2.2.

## Results

A total of 4836 survey respondents reported whether they consumed alcohol within the last 12 months, 30.6% (95% CI 29.3–31.9%) of whom did not consume alcohol (including lifetime abstainers), 57.4% (95% CI 56.0–58.8%) had consumed alcohol a few days per month or less frequently, and 11.6% (95% CI 10.7–12.6%) indicated that during the past 12 months they consumed alcohol at least once per week. According to the survey protocol, the latter group (*n* = 562) was further surveyed for the alcohol frequency and quantity habits during the week.

After applying the indicated individual proportions of unrecorded consumption for weekly drinkers, it was estimated that overall 8.3% (95% CI 7.7–8.9%) of total alcohol consumption reported by the respondents in 2019 was unrecorded. Out of this, 3.49% (95% CI 3.1–3.8%) was attributed to unrecorded beer consumption, 3.39% (95% CI 3.06–3.72%) to unrecorded strong alcohol beverages like vodka, 1.36% (95% CI 1.15–1.57%) to unrecorded wine, and the remaining 0.06% (95% CI 0.02–0.10%) was attributed to cocktails and fermented drinks as being unrecorded.

Because the total recorded alcohol consumption in 2019 was 11.2 L and the unrecorded consumption being estimated at 1.01 L in 2019, we believe the total consumption likely to be 12.2 L of pure alcohol per 15 years or older population. The summary of the results is provided in Table 3.

**Table 3:** Estimates of unrecorded APC consumption and total APC consumption in Lithuania (litres of pure alcohol among 15 years old and older population), 2000–2021.

Year	APC estimates based on K70 and X45				APC estimates based on majority of fully alcohol attributable mortality (K70, X45, F10, G31.2, G62.1, I42.6, K86.0, X65, Y15)			
	Relative changes of unrecorded alcohol consumption, 2019 = 100	Unrecorded APC, litres of pure alcohol among 15+ population	Proportion of unrecorded alcohol, %	Total (recorded and unrecorded) APC	Relative changes of unrecorded alcohol consumption, 2019 = 100	Unrecorded APC, litres of pure alcohol among 15+ population	Proportion of unrecorded alcohol, %	Total (recorded and unrecorded) APC)
2000	92.42	0.94	8.80%	10.64				
2001	110.48	1.12	9.63%	11.62				
2002	92.31	0.94	7.77%	12.04	83.67	0.85	7.04%	11.95
2003	106.95	1.08	8.75%	12.38	99.13	1.00	8.11%	12.30
2004	85.81	0.87	6.65%	13.07	82.76	0.84	6.41%	13.04
2005	112.54	1.14	8.36%	13.64	101.59	1.03	7.54%	13.53
2006	109.65	1.11	7.76%	14.31	103.38	1.05	7.32%	14.25
2007	105.97	1.07	7.17%	14.97	101.46	1.03	6.86%	14.93
2008	85.51	0.87	5.87%	14.77	77.90	0.79	5.34%	14.69
2009	75.87	0.77	5.54%	13.87	68.35	0.69	4.99%	13.79
2010	86.74	0.88	6.07%	14.48	83.86	0.85	5.87%	14.45
2011	90.32	0.91	5.78%	15.82	81.56	0.83	5.22%	15.73
2012	91.56	0.93	5.76%	16.10	86.72	0.88	5.46%	16.05
2013	101.90	1.03	6.37%	16.19	89.49	0.91	5.60%	16.07
2014	80.74	0.82	5.22%	15.67	78.71	0.80	5.09%	15.65
2015	100.64	1.02	6.58%	15.48	90.80	0.92	5.94%	15.38
2016	93.96	0.95	6.52%	14.59	91.43	0.93	6.35%	14.57
2017	98.46	1.00	7.46%	13.37	90.20	0.91	6.83%	13.29
2018	114.67	1.16	9.33%	12.45	106.09	1.07	8.63%	12.36
**2019** ^**a**^	**100.00**	**1.01**	**8.30%**	**12.21**	**100.00**	**1.01**	**8.30%**	**12.21**
2020	108.14	1.10	8.74%	12.53	94.34	0.96	7.63%	12.39
2021	100.44	1.02	7.73%	13.16	95.17	0.96	7.33%	13.10

^a^Reference year for estimates of unrecorded APC in Lithuania.

According to these estimates, the lowest unrecorded alcohol levels were in 2009 and 2014, when it was 0.77 L per capita (5.54% of total APC) and 0.82 L per capita (5.22%), respectively. We estimated the highest level of unrecorded alcohol for the Year 2018, when it reached 1.16 L per capita (9.33% of total APC). Another estimate, based on an alternate scenario in which 100% of the possible causes of death are assumed to be attributed to alcohol, yields very similar results as evidenced by the perfectly correlated predictions (Pearson correlation: 0.98; df = 20; *P* < .001).

## Discussion

This study provides unrecorded alcohol consumption estimates for Lithuania based on the 2019 EHIS survey data. The results indicate that 8.3% of total APC consumption was unrecorded in 2019. An additional indirect analysis of alcohol poisonings and alcoholic liver cirrhosis mortality was applied to assess a change in total alcohol consumption (recorded and unrecorded consumption) for over two decades. Overall, these estimates fluctuate between 5.22% at the lowest and 9.33% at the highest, indicating that Lithuania had a relatively low absolute level of unrecorded consumption in comparison to high level of recorded consumption.

According to the annual report by Orro and colleagues, unrecorded alcohol consumption in 2020 in Finland and Sweden may have been around 12%, while in Norway 10%. In contrast, in the much smaller country of Estonia, the tourist exports, alcohol consumption by tourists, alcohol bought abroad, and illegal sales seem to cancel each other out, resulting in a relatively low net effect on total alcohol consumption (Estonian Institute of Economic Research 2021). This is all to say that there is a bit of country-specific idiosyncrasy with regard to unrecorded consumption, but Lithuanian estimates are overall similar to other Baltic Sea Region states well-known for their relatively strictly regulated alcohol policy, and the EU context such as free movement across Schengen’s area countries.

The level of unrecorded APC in Lithuania is relatively low, and the fluctuations may well be random. Therefore, the high level of recorded alcohol consumption in Lithuania continues as the main target for the public health policy response. In 2015–2018, the recorded APC has declined by over 3 L of pure alcohol, followed by more stagnant APC level between 2018 and 2020, and increasing to over 12 L in 2021. This increase is paralleled by increases in 100% alcohol-attributable causes of death, indicating that a new round of alcohol control policy may be needed.

The changes of total alcohol consumption overlap with the recent changes in alcohol control policies in Lithuania when significant increases in alcohol excise tax were introduced in 2017, with additional availability restrictions coming into effect in 2018, reducing retail hours and increasing legal age of purchase (Miščikienė *et al.* 2020). The policy effect was also evident in many other indicators of alcohol-related harm in Lithuania (Rehm *et al.* 2020, Lange *et al.* 2021, Radisauskas *et al.* 2021, Štelemėkas *et al.* 2021), and overall alcohol consumption has been significantly reduced with implementation of the ‘best buy’ policies in the Baltic countries and Poland (Rehm *et al.* 2022b).

The question regarding unrecorded alcohol consumption in EHIS survey asked very generally about respondents' overall unrecorded alcohol consumption—including legal cross-border shopping and illegal consumption. Therefore, it should not have triggered any additional concerns for the respondents over whether to be dishonest (i.e., minimizing the indicated proportion) when answering it.

One of the important limitations of this study comes from the EHIS survey protocol when only the most alcohol-consuming group—i.e. weekly drinkers—were further probed for frequency and quantity of alcohol consumption, while these questions were omitted if the respondents indicated that they drank ‘a few days per month or less frequently’. In the current analysis, we assumed that lighter drinkers consume the same proportion of unrecorded alcohol as heavier drinkers. The second limitation inherent to the EHIS survey is that it may under-sample heavy drinkers. The rates of current abstainers of 30% (even though primarily driven by 65 and older population group) and weekly alcohol users of 10% as estimated in EHIS are deviating from survey results such as RARHA SEAS (Moskalewicz *et al.* 2016): <10% current abstainers.

Another limitation of the presented findings stems from the instability of the time series models. The main principle of the Norström technique (Norström 1998) is that ‘excess error’ in estimating the relation between mortality and recorded alcohol consumption is attributed to unrecorded alcohol consumption. If time series models had errors equal to zero, the Norström technique would estimate the unrecorded alcohol consumption perfectly. However, in the real-world, this is never the case, and is one of the main ways that the Norström technique might fail to estimate variations in unrecorded alcohol accurately, both by over- and underestimation. This is one of the reasons why this method is suggested to be as a supplement to the survey-based methodologies to estimate the volume of unrecorded consumption over time. Lastly, the time series of both recorded alcohol consumption and mortality rates must be sufficiently long to form a reliable model. There are mainly rules of thumb about minimum length (Beard *et al.* 2019, Jiang *et al.* 2022), which indicate that our study has just enough time points needed for a *lege-artis* time series analysis.

Despite the current limitations indicated above, this study presents an important insight in an under researched area about the unrecorded alcohol consumption in Lithuania. The EHIS survey should be considered and explored in future studies as an important routine European tool for a potential to monitor alcohol consumption (recorded and unrecorded) as for smaller countries like Lithuania it may be the only standardized survey to collect the beverage-specific quantity and frequency data with an addition of unrecorded alcohol consumption component.

The results of this article may serve as an illustration for the European Institutions organizing EHIS about the need to include any alcohol-consuming respondents into the full set of questions regarding alcohol consumption. After all, the surveys serve as one of the main methods in studying unrecorded alcohol consumption in a population, while expert surveys are another method applicable in situation when the population survey data are unavailable (Probst *et al.* 2019).

In conclusion, this study illustrates that unrecorded alcohol consumption in Lithuania is likely to be at a relatively modest level compared to recorded alcohol consumption, suggesting that recorded consumption remains the major challenge to public health. The indirect methodology applied to estimate the annual change in unrecorded alcohol consumption also suggests that unrecorded alcohol consumption has remained at a relatively constant level. This approach may be a valuable supplementary and cost-effective methodology, in addition to a survey-based estimates of time changes of unrecorded alcohol consumption.

## Supplementary Material

2022_12_19_Unrecorded_alcohol_in_Lithuania_SUPPLEMENT_agad063

## Data Availability

The data may be obtained on request through the Lithuanian Governmental institutions (Lithuanian Institute of Hygiene, Statistics Lithuania).

## References

[ref1] Babor TF, Casswell S, Graham K et al. Alcohol: No Ordinary Commodity, III edn, Oxford University Press, 2023.

[ref2] Beard E, Marsden J, Brown J et al. Understanding and using time series analyses in addiction research. Addiction 2019;114:1866–84. 31058392 10.1111/add.14643

[ref3] EHIS . European Health Interview Survey (EHIS Wave 3) — Methodological Manual. Luxembourg: Publications Office of the European Union, 2018. https://ec.europa.eu/eurostat/web/products-manuals-and-guidelines/-/ks-02-18-240 (2 December 2022, date last accessed).

[ref4] Estonian Institute of Economic Research . Alcohol Market, Consumption and Harms in Estonia. Yearbook 2021. Tallinn: Health Development Institute, Estonian Economic Institute, 2021. https://www.tai.ee/et/valjaanded/alkoholi-turg-tarbimine-ja-kahjud-eestis-aastaraamat-2021-alcohol-market-consumption-and (26 November 2022, date last accessed).

[ref5] Eurostat . European Health Interview Survey (EHIS). 2020. https://ec.europa.eu/eurostat/web/microdata/european-health-interview-survey (25 November 2022, date last accessed).

[ref6] Grabauskas V, Prochorskas R, Veryga A. Associations between mortality and alcohol consumption in Lithuanian population. *Medicina* (*Kaunas*) 2009;45:1000–12. 20173404

[ref7] Health Research Institute . Estimates of Unrecorded Alcohol in Lithuania in 2016–2017 [Neapskaityto Alkoholio Suvartojimo Lietuvoje į Vertinimas 2016–2017 M.]. A Report for the Department of Drug Tobacco and Alcohol Control. 2018. https://ntakd.lrv.lt/uploads/ntakd/documents/files/Neapskaitytos%20alkoholio%20apyvartos%20vertinimas%202018_12_20%20(galutinis).pdf (24 November 2022, date last accessed).

[ref8] Institute of Hygiene . Health Statistics Data Portal [Sveikatos statistinių duomenų Portalas]. 2022. https://www.hi.lt/sveikatos-statistika.html (25 November 2022, date last accessed).

[ref9] Jiang H, Feng X, Lange S et al. Estimating effects of health policy interventions using interrupted time-series analyses: a simulation study. BMC Med Res Methodol 2022;22:235. 36045338 10.1186/s12874-022-01716-4 PMC9429656

[ref10] Lachenmeier DW, Neufeld M, Rehm J. The impact of unrecorded alcohol use on health: what do we know in 2020? J Stud Alcohol Drugs 2021;82:28–41. 33573720

[ref11] Lange S, Jiang H, Štelemėkas M et al. Evaluating the impact of alcohol policy on suicide mortality: a sex-specific time-series analysis for Lithuania. Arch Suicide Res 2021;27:339–352. 10.1080/13811118.2021.1999873 PMC9098693 34779348

[ref12] LRT . WHO Data: Lithuanians Are Still First - they Consume 16.3 Liters of Alcohol [Media Article in Lithuanian: PSO Duomenys: Lietuviai vis Dar Pirmi – Suvartoja 16,3 Litro Alkoholio]. 2017. *lrt.lt*. https://www.lrt.lt/naujienos/lietuvoje/2/176472/pso-duomenys-lietuviai-vis-dar-pirmi-suvartoja-16-3-litro-alkoholio (1 December 2022, date last accessed).

[ref13] Manthey J, Probst C, Kilian C et al. Unrecorded alcohol consumption in seven European Union countries. EAR 2020;26:316–25 Karger Publishers. 10.1159/000506333 32114584

[ref14] Miščikienė L, Goštautaitė Midttun N, Galkus L et al. Review of the Lithuanian alcohol control legislation in 1990–2020. Int J Environ Res Public Health 2020;17:3454 Multidisciplinary Digital Publishing Institute. 32429171 10.3390/ijerph17103454 PMC7277450

[ref15] Moskalewicz J, Room R, Thom B. Comparative Monitoring of Alcohol Epidemiology across the EU: Baseline Assessment and Suggestions for Future Action, Joint Action on Reducing Alcohol Related Harm (RARHA) report. PARPA – The State Agency for Prevention of Alcohol Related Problems, Warsaw, 2016.

[ref16] Norström T . Estimating changes in unrecorded alcohol consumption in Norway using indicators of harm. Addiction 1998;93:1531–8. 9926557 10.1046/j.1360-0443.1998.931015319.x

[ref17] Probst C, Fleischmann A, Gmel G et al. The global proportion and volume of unrecorded alcohol in 2015. J Glob Health 2019;9:010421. 31131099 10.7189/jogh.09.010421 PMC6513411

[ref18] Radisauskas R, Kim KV, Lange S et al. Cardiovascular diseases mortality and alcohol control policy in Lithuania: exploring a possible link. BMC Public Health 2021;21:2116. 34789207 10.1186/s12889-021-12177-7 PMC8600709

[ref19] Rehm J, Manthey J, Lange S et al. Alcohol control policy and changes in alcohol-related traffic harm. Addiction 2020;115:655–65. 31475395 10.1111/add.14796

[ref20] Rehm J, Neufeld M, Room R et al. The impact of alcohol taxation changes on unrecorded alcohol consumption: a review and recommendations. Int J Drug Policy 2022a;99:103420. 34456119 10.1016/j.drugpo.2021.103420 PMC9429812

[ref21] Rehm J, Tran A, Gobiņa I et al. Do alcohol control policies have the predicted effects on consumption? An analysis of the Baltic countries and Poland 2000–2020. Drug Alcohol Depend 2022b;241:109682. 36402051 10.1016/j.drugalcdep.2022.109682 PMC9772294

[ref22] Sama TB, Konttinen I, Hiilamo H. Alcohol industry arguments for liberalizing alcohol policy in Finland: analysis of twitter data. J Stud Alcohol Drugs 2021;82:279–287; Rutgers University. 33823975

[ref23] Statistics Lithuania . Methodology to Estimate Recorded Alcohol Consumption [Legalaus Alkoholio Suvartojimo skaičiavimo aprašas]. Order of the Director of Statistics Lithuania DĮ-125. 2016. https://www.stat.gov.lt/documents/10180/5118910/Alkoholio%2C+tabako+vartojimas+ir+padariniai+%5BLT%5D+16.html (24 November 2022, date last accessed).

[ref24] Statistics Lithuania . Offical Stastics Portal (Lithuania). Health of the Lithuanian Population: Alcohol Consumption 2020. 2020. https://osp.stat.gov.lt/lietuvos-gyventoju-sveikata-2020/alkoholio-vartojimas (24 November 2022, date last accesed).

[ref25] Statistics Lithuania . Official Statistics Portal. 2022. https://osp.stat.gov.lt/statistiniu-rodikliu-analize#/ (24 November 2022, date last accessed).

[ref26] Štelemėkas M, Manthey J, Badaras R et al. Alcohol control policy measures and all-cause mortality in Lithuania: an interrupted time-series analysis. Addiction 2021;116:2673–84. 33751693 10.1111/add.15470 PMC8873029

[ref27] Stumbrys D, Telksnys T, Jasilionis D et al. Alcohol-related male mortality in the context of changing alcohol control policy in Lithuania 2000-2017. Drug Alcohol Rev 2020;39:818–826. 10.1111/dar.13059 32196816

[ref28] WHO . Global Status Report on Alcohol and Health 2018. Geneva: World Health Organization, 2018, 2018. Licence: CC BY-NC-SA 3.0 IGO.

[ref29] Zatonski M, Hawkins B, McKee M. Framing the policy debate over spirits excise tax in Poland. Health Promot Int 2018;33:515–24. 28011653 10.1093/heapro/daw093 PMC6005036

