# Supplementary material for: Unrecorded alcohol consumption in Lithuania: a modelling study for 2000–2021
Source: Alcohol Alcohol. 2023 Oct 7;58(6):612–8. doi: 10.1093/alcalc/agad063 (PMC10642605; doi:10.1093/alcalc/agad063)
Supplement: 2022_12_19_Unrecorded_alcohol_in_Lithuania_SUPPLEMENT_agad063 [file 2022_12_19_unrecorded_alcohol_in_lithuania_supplement_agad063.docx]

## Supplement. Translated alcohol related survey questions from the Lithuanian EHIS survey: V. Alcohol consumption.

**V1. In the past 12 months, how often have you had an alcoholic drink of any kind [beer, wine, cider, spirits, cocktails, premixes, liquor, homemade alcohol…]?**

1. Every day or almost

2. 5 - 6 days a week

3. 3 - 4 days a week

4. 1 - 2 days a week

5. 2 - 3 days in a month

6. Once a month

7. Less than once a month

8. Never during the past 12 months, as gave up alcohol completely

9. Never or only tasted it a few times in your life

**V2. Thinking of Monday to Thursday, on how many of these 4 days do you usually drink alcohol?**

1. On all 4 days

2. On 3 of the 4 days

3. On 2 of the 4 days

4. On 1 of the 4 days

5. On none of the 4 days

**V3. From Monday to Thursday, how many drinks do you have on average on such a day when you drink alcohol? (If you drink beer one day and wine or spirits the next day, enter the amount of the most commonly consumed alcoholic drink, choosing the usual volume unit for you)**

Number

| beer | 1 | glass | 200 ml |  |
| --- | --- | --- | --- | --- |
|  | 2 | can, mug | 0,33 l |  |
|  | 3 | can, mug, bottle | 0,5 l |  |
| wine, sparkling wine | 4 | glass | 100 ml |  |
|  | 5 | glass | 200 ml |  |
|  | 6 | bottle | 750 ml |  |
| spirits | 7 | shot | 30-40 ml |  |
|  | 8 | shot | 50 ml |  |
|  | 9 | other | 100 ml |  |
| cocktails | 10 | glass | 100 ml |  |
|  | 11 | can, bottle | 0,33 l |  |
| fermented drinks | 12 | glass | 100 ml |  |
|  | 13 | can. bottle | 0,33 l |  |
|  | 14 | can | 0,5 l |  |

**V4. Thinking of Friday to Sunday, on how many of these 3 days do you usually drink alcohol?**

1. On all 3 days

2. On 2 of the 3 days

3. On 1 of the 3 days

4. On none of the 3 days

**V5. From Friday to Sunday, how many drinks do you have on average on such a day when you drink alcohol? (If you drink beer one day and wine or spirits the next day, enter the amount of the most commonly consumed alcoholic drink, choosing the usual volume unit for you)**

Number

| beer | 1 | glass | 200 ml |  |
| --- | --- | --- | --- | --- |
|  | 2 | can, mug | 0,33 l |  |
|  | 3 | can, mug, bottle | 0,5 l |  |
| wine, sparkling wine | 4 | glass | 100 ml |  |
|  | 5 | glass | 200 ml |  |
|  | 6 | bottle | 750 ml |  |
| spirits | 7 | shot | 30-40 ml |  |
|  | 8 | shot | 50 ml |  |
|  | 9 | other | 100 ml |  |
| cocktails | 10 | glass | 100 ml |  |
|  | 11 | can, bottle | 0,33 l |  |
| fermented drinks | 12 | glass | 100 ml |  |
|  | 13 | can. bottle | 0,33 l |  |
|  | 14 | can | 0,5 l |  |

**V6. During the past 12 months, how often there have been occasions when you drank 1.5 litres or more of beer or 6 glasses (600 ml) or more of wine or 5 glasses (200 ml) or more of vodka, or other hard liquor on one occasion? (E.g. at a party, having dinner, entertaining with friends or at home alone, etc.)**

1. Every day or almost

2. 5 - 6 days a week

3. 3 - 4 days a week

4. 1 - 2 days a week

5. 2 - 3 days in a month

6. Once a month

7. Less than once a month

8. Not in the past 12 months

9. Never

**V7. What proportion of the total consumed alcohol during the past 12 months, consisted of alcoholic beverages purchased in stores in other countries (Latvia, Poland, etc.) and brought back to Lithuania and/or home-made (home-made wine, homemade vodka) and/or non-industrially produced drinks (surrogate alcohol, diluted spirits)? (If you only consumed beverages legally purchased in Lithuania, enter 0.)**

% of the consumed amount of this type of drink

| 1 | beer |  |
| --- | --- | --- |
| 2 | wine, sparkling wine |  |
| 3 | spirits |  |
| 4 | cocktails |  |
| 5 | fermented drinks |  |
